# KGLA-Net: an efficient facial affective computing framework for psychomotor phenotyping in psychiatry

**DOI:** 10.3389/fpsyt.2026.1903055

**Published:** 2026-09-04

**Authors:** Fengze Li, Jiaying Yu, Minglei Sun, Xiang Li

**Affiliations:** School of Industrial Design, Lu Xun Academy of Fine Arts, Liaoning, Shenyang, China

**Keywords:** dual-stream network, facial expression recognition, local aggregation, product design, psychomotor phenotyping

## Abstract

Facial Expression Recognition (FER) has emerged as an important affective phenotyping tool in computational psychiatry, providing objective measurements of facial expressivity associated with psychomotor abnormalities in psychiatric disorders. From the perspective of smart product design, deploying deep learning frameworks for longitudinal psychiatric monitoring requires balancing recognition accuracy with hardware constraints. Since existing dual-stream networks often rely on computationally intensive global cross-attention, their integration into resourceconstrained products or interactive terminals remains challenging. To address these limitations, we present KGLA-Net, a dual-stream network. Our framework extracts parallel representations through a landmark backbone and an image backbone across three hierarchical stages. In the structural stream, a feature refinement module processes emotion-relevant cues, which are then aggregated through max-pooling to form multi-scale refined keypoint features. This structural prior drives a Keypoint-Guided Local Aggregation (KGLA) module, employing multi-head cross attention across localized multi-scale windows (8 × 8, 4 × 4, and 2 × 2) to guide the aggregation of image features, thereby reducing the computational complexity to linear complexity. Finally, the aggregated features are processed via keypoint feature self-attention before interacting with a zero-initialized query in stacked attention blocks. Experimental evaluations demonstrate that KGLA-Net achieves competitive FER performance while maintaining high computational efficiency. The proposed framework provides an efficient solution for facial affective computing and offers a lightweight foundation for future research on computational psychiatry.

## 1 Introduction

Facial Expression Recognition (FER) has established itself as a fundamental task in computer vision, unlocking profound interdisciplinary applications (1–3). In clinical psychiatry, psychiatric disorders are frequently accompanied by psychomotor abnormalities that affect both emotional expression and motor behavior (4, 5). As objective behavioral analysis has become an important direction in computational psychiatry, facial affective computing has emerged as a practical and non-invasive approach for psychomotor phenotyping. From the perspective of human-centered smart product design, deploying FER models for long-term psychiatric monitoring requires not only high recognition accuracy but also computational efficiency for resource-constrained intelligent devices and healthcare terminals. Therefore, developing lightweight yet robust FER algorithms is essential for facilitating real-world affective computing applications, complementing recent advances in gait-based emotion recognition (6).

The primary challenges in real-world FER lie in mitigating inter-class similarity and intra-class discrepancy (7, 8). Expressions like sadness and fear often share overlapping muscle activations, while expressions from different identities present vast visual variations. Recent studies have addressed these challenges by adopting dual-stream architectures that leverage both facial appearances and facial landmarks (9, 10). Because landmarks provide a sparse structural representation of the face, they serve as geometric priors to guide the network’s attention toward expression-salient regions while suppressing irrelevant visual variations. Representative landmark-guided dual-stream methods are reviewed in detail in Section 2. Nevertheless, most existing methods still struggle with two major bottlenecks: first, they often compute cross-modal guidance using vanilla global cross-attention (11), which intrinsically suffers from quadratic computational complexity; second, their multi-scale feature integration strategies can be disjointed, failing to efficiently propagate refined keypoint semantics into hierarchical visual feature maps.

To alleviate these computational and architectural bottlenecks, we propose KGLA-Net, an efficient dual-stream architecture centered on keypoint-guided cross-modal aggregation. Unlike POSTER and POSTER++, which repeatedly perform cross-modal fusion between hierarchical landmark and image features, KGLA-Net first constructs a unified structural prior and subsequently reuses it to guide progressive cross-modal interaction. Specifically, facial landmarks and RGB images are processed by a Landmark Backbone and an Image Backbone, respectively, to extract hierarchical structural and visual representations. The landmark features are then refined by a Feature Refinement Module (FRM) and integrated into a unified multi-scale structural prior that serves as the guidance for subsequent feature aggregation. Based on this structural prior, we introduce the proposed Keypoint-Guided Local Aggregation (KGLA) module. Instead of performing computationally expensive global cross attention, KGLA conducts multi-head cross attention within dynamically partitioned local windows at multiple scales, allowing structural priors to selectively aggregate spatially corresponding visual features. This localized cross-modal interaction helps preserve fine-grained expression information while reducing the computational complexity from quadratic to linear. Finally, the aggregated visual features are further refined through Keypoint Feature Self-Attention to enhance global contextual dependencies. An initial learnable query is subsequently employed to progressively interact with the aggregated features through stacked attention blocks, and the resulting representation is fed into a classification head for facial expression recognition.

In summary, the main contributions of our work are:

- We underscore the universal value of efficient FER algorithms by highlighting their contactless sensing potential in both human-centric industrial design research and psychiatric affective phenotyping.
- We propose KGLA-Net, an efficient dual-stream architecture. Within this network, we design a multiscale refined keypoint feature strategy that distills and unifies hierarchical landmark features into a powerful, identity-agnostic structural prior.
- We develop a Keypoint-Guided Local Aggregation (KGLA) mechanism that performs localized cross-modal aggregation under structural guidance.
- We introduce an elegant query-based classification head that iteratively decodes the keypoint-guided aggregated features. Extensive experiments demonstrate that KGLA-Net achieves state-of-the-art accuracy with superior efficiency compared to existing complex dual-stream networks.

## 2 Related works

### 2.1 Facial expression recognition

The study of Facial Expression Recognition (FER) has attracted significant attention as a core component of human-computer interaction. Early FER research primarily relied on hand-crafted features, such as Local Binary Patterns (LBP) introduced by Zhao et al. (12), to analyze facial expressions. However, these manual representations often lack robustness when applied to diverse, real-world scenarios. With the advent of deep learning, Convolutional Neural Networks (CNNs) were introduced to improve model robustness. For instance, Sang et al. (13) proposed a dense convolutional network to reduce intra-class variation, and Mohan et al. (14) introduced FER-net with a softmax classifier for efficient expression distinction. While these CNN-based methods enhanced FER performance, their inherent reliance on local receptive fields prevented them from fully capturing the global structure of facial images. To overcome the limitations of CNNs, vision transformers have been introduced to model long-range dependencies. Xue et al. (7) were the first to employ a vision transformer for FER research, achieving state-of-the-art performance. Kim et al. (15) further improved the vision transformer to combine both global and local features. Despite these advancements, robust FER continues to face three key challenges: inter-class similarity, intra-class discrepancy, and scale sensitivity. Furthermore, deploying computationally heavy models for continuous monitoring in psychiatry or real-time user experience evaluation in industrial design remains challenging, as also evidenced in recent emotion perception studies (16, 17).

### 2.2 Dual-stream networks and landmark-guided fusion

To address the critical issues of intra-class variation and inter-class similarity, recent works have extensively explored fusing multiple features or modalities (18–22). For instance, FS-DSN (19) presents a feature segmentation-based dual-stream network to capture robust representations in uncontrolled environments, highlighting the potential of multi-stream feature alignment. Similarly, FLEPNet (23) focuses on fusing combined features retrieved by parallel networks to reduce intra-class differences. More recently, incorporating facial landmarks has been shown to be effective. Landmarks provide a sparse, identityagnostic structural prior that intuitively directs the network to focus on expression-salient regions while disregarding identity-specific textures. A prominent milestone in this direction is POSTER (9), which simultaneously addressed intra-class discrepancy, inter-class similarity, and scale sensitivity by integrating image features with facial landmark features through a two-stream, cross-fusion, and pyramid design. However, POSTER’s architecture involves a huge number of parameters and an expensive computational cost, primarily due to its vanilla cross-attention mechanism which operates with square-level computational complexity. To resolve this, POSTER++ (10) simplified the two-stream design and introduced a multiscale interaction network to replace the pyramidal architecture, significantly improving efficiency. While POSTER++ optimized the global architecture, its cross-modal fusion still leaves room for fine-grained local alignment. In contrast, our proposed KGLA-Net utilizes multi-scale refined keypoint features to guide the visual stream locally, effectively bridging structural priors and visual textures without prohibitive computational costs. More importantly, unlike POSTER++, which directly employs hierarchical landmark features for repeated cross-modal interactions, KGLA-Net first constructs a unified structural prior and then reuses this prior throughout progressive local aggregation.

### 2.3 Efficient vision transformers and local attention

Vision transformers (11, 24) have been widely used for computer vision tasks due to their excellent ability to model long-range dependencies. To adapt transformers to different scales while reducing computational cost, several efficient architectures have been proposed. Yuan et al. (25) introduced T2T-ViT to progressively aggregate neighboring tokens for more effective visual representation learning. Chen et al. (26) proposed CrossViT, which employs dual branches with different patch sizes to capture multiscale features. Wang et al. (27) presented Pyramid Vision Transformer (PVT), introducing the Spatial Reduction Attention (SRA) mechanism that reduces the spatial resolution of keys and values before attention computation, thereby significantly lowering computational complexity. Liu et al. (28) further proposed the Swin Transformer, which adopts shifted local windows to reduce the quadratic complexity of global self-attention while preserving hierarchical feature representations. These efficient attention mechanisms have inspired the design of KGLA-Net. Different from SRA, which reduces computational complexity by spatially downsampling feature maps, and Swin Transformer, which performs localized selfattention within image windows, the proposed Keypoint-Guided Local Aggregation (KGLA) introduces structural priors into localized cross-modal interactions. By performing multi-scale cross attention within dynamically partitioned local windows, KGLA effectively injects landmark semantics into hierarchical visual representations while maintaining linear computational complexity.

### 2.4 State space models for facial expression recognition

Recently, State Space Models (SSMs) have emerged as an efficient alternative to Transformer-based architectures for long-range dependency modeling. Unlike self-attention, whose computational complexity grows quadratically with the sequence length, SSMs model long-range dependencies through state transition mechanisms, enabling linear computational complexity while preserving strong global representation capability. Vision Mamba (29) first introduced bidirectional state-space modeling into visual representation learning, demonstrating that competitive visual representations can be achieved with substantially improved computational efficiency. Building upon this idea, EfficientViM (30) further enhanced inference efficiency by introducing an efficient hidden-state mixer, making SSM-based architectures increasingly attractive for resource-constrained vision applications. Motivated by these advances, recent FER methods have begun incorporating Mamba-based architectures into facial expression recognition. FERMam (31) integrates CNNs, Mamba, and facial landmark information through a lightweight dual-source multi-scale fusion framework, effectively balancing recognition accuracy and computational cost. Similarly, Mambaer (32) combines CNNs, Mamba, and Transformers to facilitate efficient multi-granularity feature interaction while alleviating the computational burden of conventional Transformer-based architectures. Different from these methods, KGLA-Net improves computational efficiency from the perspective of cross-modal interaction rather than backbone design. Instead of replacing the visual backbone with an SSM-based architecture, the proposed Keypoint-Guided Local Aggregation (KGLA) transforms global cross-attention into localized cross-modal aggregation with linear complexity, enabling efficient structural prior propagation while preserving discriminative visual representations. Therefore, our method is complementary to recent SSMbased FER architectures and could potentially benefit from future integration with Mamba-based visual backbones.

## 3 Methods

### 3.1 Overall architecture

**Figure 1:**
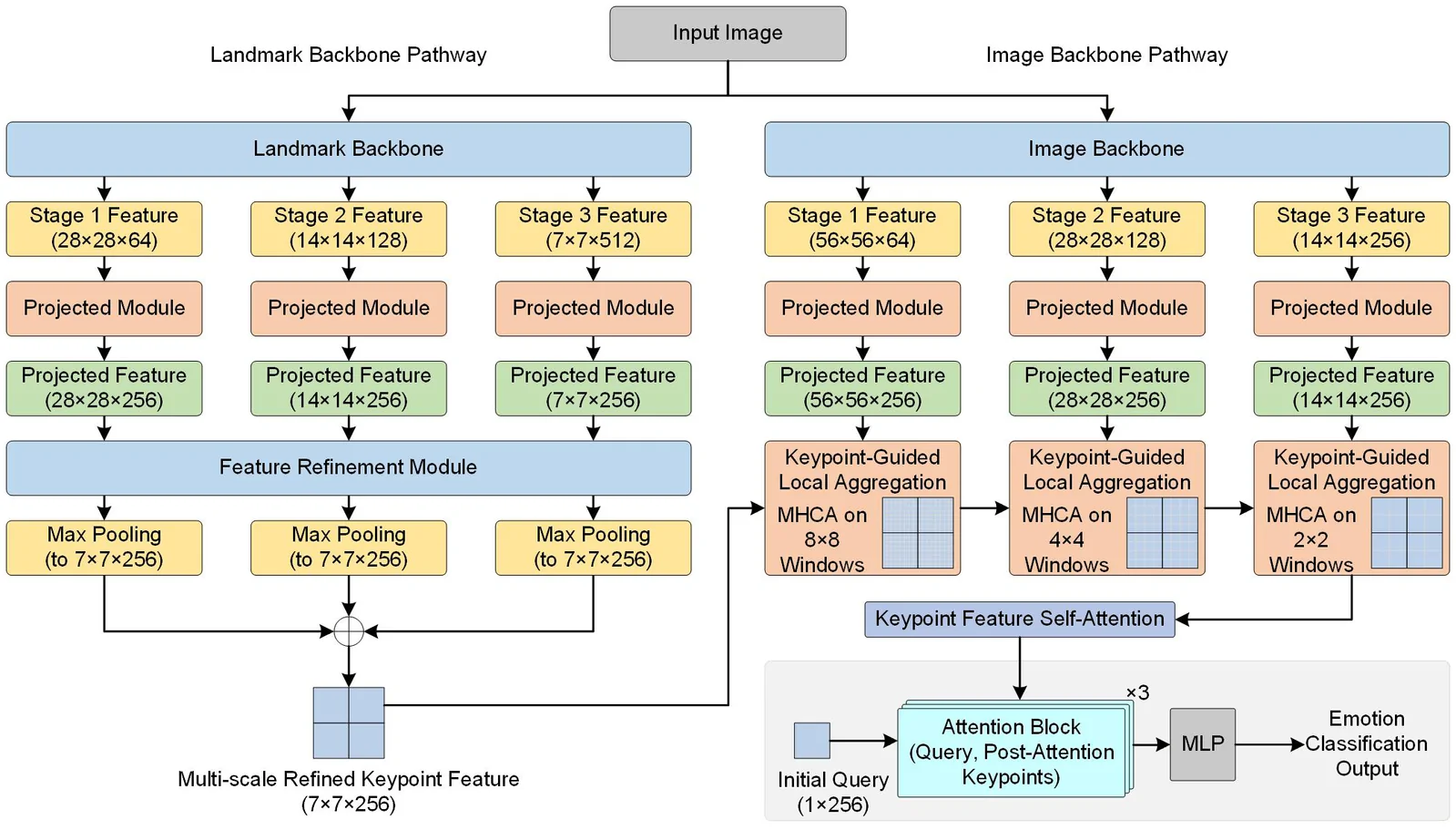
The overall architecture of the proposed KGLA-Net. The network consists of a Landmark Backbone and an Image Backbone. Features from three stages are projected to a unified channel dimension. The structural features are refined and pooled to generate a Multi-scale Refined Keypoint Feature. This structural prior is then used to guide the aggregation of image features via Multi-Head Cross Attention (MHCA) across shrinking local windows (8×8, 4×4, and 2×2). Finally, Keypoint Feature Self-Attention and an all-zero Initial Query are employed in stacked Attention Blocks to predict the emotion class.

The overall architecture of the proposed Keypoint-Guided Local Aggregation Network (KGLA-Net) is illustrated in **Figure 1**. To address the challenges of inter-class similarity and intra-class discrepancy in Facial Expression Recognition (FER), KGLA-Net is designed to efficiently extract, refine, and aggregate cross-modal features. The pipeline consists of four main stages: 1) Dual Backbone Extraction & Projection, which extracts multi-scale spatial and visual features; 2) Keypoint Refinement & Multi-scale Fusion, which distills the structural prior; 3) Keypoint-Guided Local Aggregation (KGLA), which locally aggregates visual textures under the guidance of keypoint semantics; and 4) Self-Attention & Emotion Classification, which decodes the aggregated features for final prediction. Compared with previous dual-stream FER frameworks, the proposed pipeline separates structural representation learning from cross-modal aggregation. The refined keypoint prior is generated only once and subsequently shared across all interaction stages, enabling more consistent structural guidance than repeated stage-wise landmark-image fusion.

### 3.2 Dual backbone extraction and projection

Given an input facial image, KGLA-Net utilizes a dual-stream architecture to independently extract structural landmarks and visual appearances. The input image is fed into a *Landmark Backbone* and an *Image Backbone* in parallel. To capture hierarchical representations, we extract features from three distinct stages (Stage 1 to Stage 3) in both backbones. Specifically, the Landmark Backbone generates spatial features with resolutions of 28 × 28 × 64, 14 × 14 × 128, and 7 × 7 × 512. Concurrently, the Image Backbone extracts visual textures with higher resolutions of 56×56×64, 28×28×128, and 14×14×256. To facilitate the subsequent cross-modal interaction, the channel dimensions of the hierarchical features from both streams must be aligned. Therefore, we introduce a *Projected Module* for each stage. These modules linearly map the channel dimensions of all six feature maps to a unified dimension of *C* = 256, producing projected landmark features (with spatial sizes 28×28, 14×14, and 7×7) and projected image features (with spatial sizes 56 × 56, 28 × 28, and 14 × 14).

### 3.3 Keypoint refinement and multi-scale fusion

**Figure 2:**
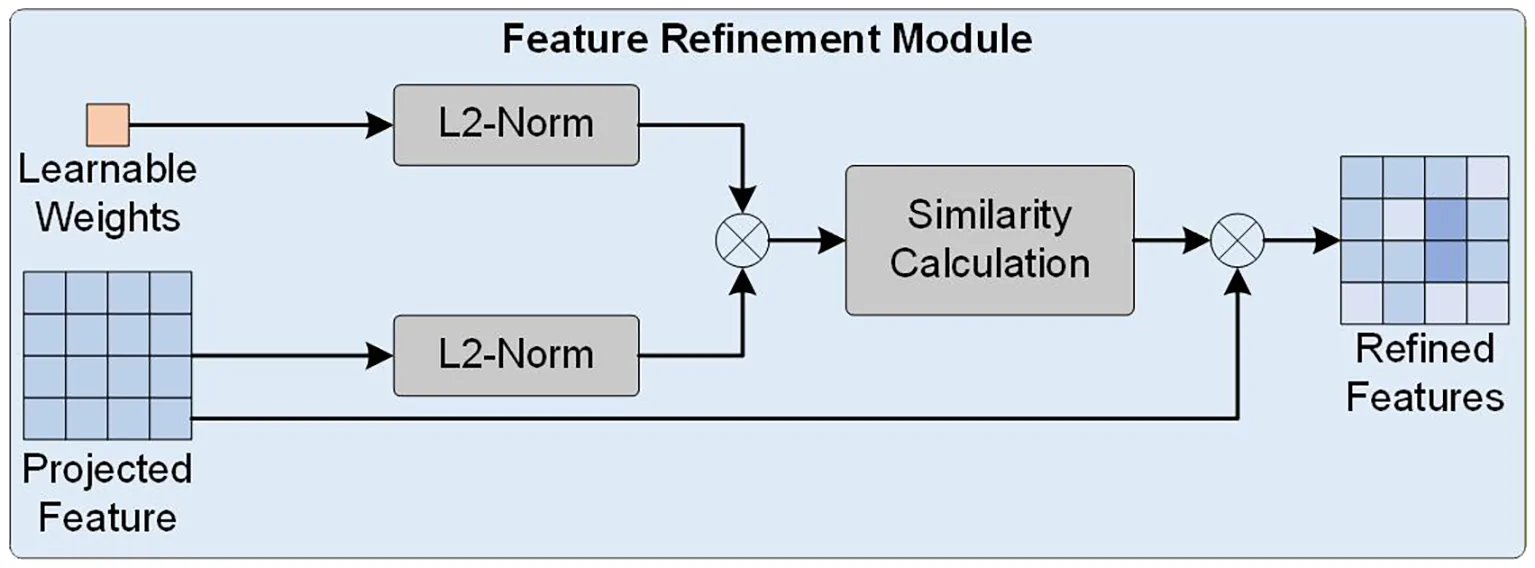
Detailed architecture of the FRM. The projected feature and the shared learnable weights are first normalized via L2-Norm. Their similarity is then evaluated through a similarity calculation function to generate a spatial refinement score map. Finally, the original projected feature is multiplied by this score map element-wise to produce the refined features.

Raw facial landmark features often contain identity-specific redundancy and background noise that are irrelevant to facial expressions. To distill pure emotional cues and suppress this noise, the projected landmark features from all three stages are fed into the FRM, as illustrated in **Figure 2**. Instead of utilizing computationally heavy linear projections, we propose a lightweight similarity scoring mechanism using a shared learnable weight vector to dynamically mask out noise points.

Let $x_{i}\in {\mathbb{R}}^{C}$ denote the feature vector of the projected landmark feature at spatial location *i*. To evaluate its relevance to facial expressions, we compute its similarity against a shared learnable weight vector $r\in {\mathbb{R}}^{C}$, whichacts as a global structural anchor. As shown in **Figure 1**, both the input feature vector *x_i_* and the shared weight *r* are first normalized using L2-Norm to obtain ${\hat{x}}_{i}$ and $\hat{r}$, respectively, as shown in Equation (1):

(1) $${\hat{x}}_{i}=\frac{x_{i}}{{\left\|x_{i}\right\|}_{2}},\;\hat{r}=\frac{r}{{\left\|r\right\|}_{2}}.$$

Subsequently, these normalized representations undergo a similarity calculation. We apply a Gaussianlike activation function to their cosine similarity ( ${\hat{x}}_{i}^{T}\hat{r}$) to generate the refinement score *S_i_*for each spatial location, as shown in Equation (2):

(2) $$S_{i}=\alpha \cdot \text{exp }\left(-\frac{{\left(1-{\hat{x}}_{i}^{T}\hat{r}\right)}^{2}}{2\sigma^{2}}\right),$$

where *α* and *σ* are learnable variables that dynamically adjust the scale and variance of the similarity distribution. This formulation ensures that features highly aligned with the structural anchor receive higher activation scores. The refined feature map is then obtained by scaling the original projected feature with the computed similarity score via element-wise multiplication, as shown in Equation (3):

(3) $$x_{i}^{\text{refined}}=S_{i}\cdot x_{i}.$$

After the refinement process, we design a multi-scale fusion strategy to construct a strong, unified structural prior. As depicted in the overall architecture, the refined features from the three stages undergo a Max Pooling operation, standardizing their spatial resolutions down to 7 × 7 × 256. Finally, these three down-sampled feature maps are aggregated via element-wise addition to generate the multi-scale refined keypoint feature ( $F_{keypoint}\in {\mathbb{R}}^{7\times 7\times 256}$). This compact tensor effectively encodes rich, identity-agnostic structural context from multiple receptive fields, preparing it to precisely guide the visual stream.

### 3.4 Keypoint-guided local aggregation

**Figure 3:**
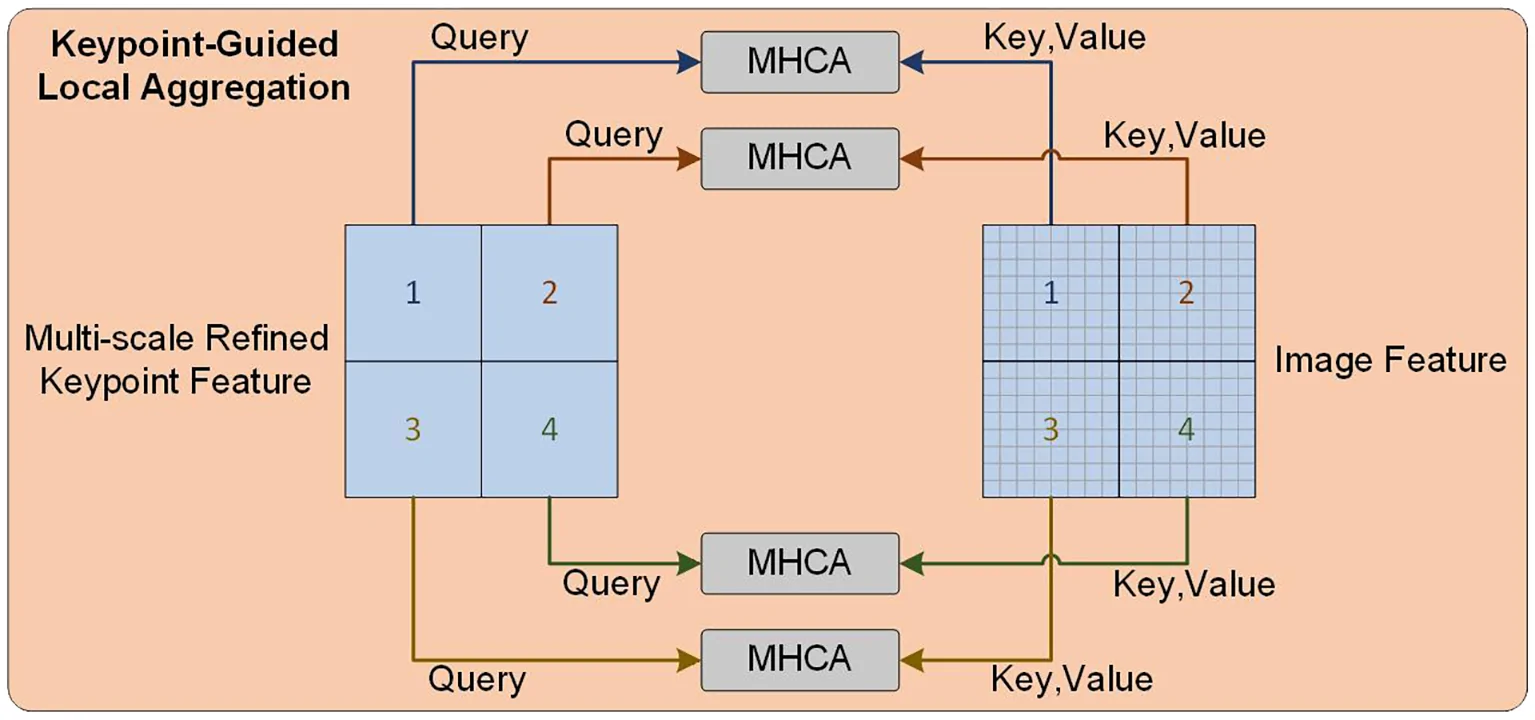
Architecture of the keypoint-guided local aggregation (KGLA) module. The multi-scale refined keypoint features and image features are spatially partitioned into localized windows. Within each corresponding region, the keypoint feature acts as the Query, while the image feature provides the Key and Value pairs for the localized Multi-Head Cross Attention (MHCA).

The generated multi-scale refined keypoint feature serves as a structural anchor to cross-modally guide the image stream. However, standard global cross-attention inherently suffers from quadratic computational complexity. To address this limitation, we introduce the Keypoint-Guided Local Aggregation (KGLA) module, which performs efficient, localized feature interactions. The motivation of localized aggregation is not solely computational efficiency. Facial expressions are primarily characterized by localized muscle activations around facial components such as the eyebrows, eyes, and mouth. Therefore, restricting crossmodal interactions to spatially corresponding regions improves semantic consistency between structural priors and visual textures while naturally eliminating redundant long-range correspondences. As illustrated in **Figure 3**, instead of computing attention globally, KGLA adaptively aggregates visual features within restricted local windows guided by the keypoint prior. We spatially partition both the refined keypoint feature and the corresponding image feature into aligned, non-overlapping local regions. Within each paired region, the local keypoint feature serves as the Query (*Q*), while the image feature provides the Key (*K*) and Value (*V)* pairs for the Multi-Head Cross Attention (MHCA) mechanism. Formally, for the *i*-th local window, the attention computation is performed as shown in Equation (4):

(4) $$\text{MHCA}\left(Q_{i},K_{i},V_{i}\right)=\text{Softmax}\left(\frac{Q_{i}K_{i}^{T}}{\sqrt{d_{k}}}\right)V_{i},$$

where *d_k_*represents the scaling factor of the feature dimension.

To enable progressive feature enhancement and maintain structural coherence, this process is cascaded across the network stages: the aggregated output feature from the preceding stage continuously serves as the Query for the subsequent stage. To accommodate the hierarchical nature of the feature maps, we employ dynamically shrinking window sizes tailored to each stage’s spatial resolution:

- For the Stage 1 image feature (56×56×256), the aggregation is restricted within 8×8 local windows.
- For the Stage 2 image feature (28×28×256), the aggregation is restricted within 4×4 local windows.
- For the Stage 3 image feature (14×14×256), the aggregation is restricted within 2×2 local windows.

This multi-scale, cascaded local window design ensures that fine-grained visual details are effectively captured under the continuous guidance of keypoint semantics across varying receptive fields, all while maintaining a linear computational complexity. To formally demonstrate this efficiency, we evaluate the computational cost in FLOPs. Let $H_{q}\times W_{q}$ be the spatial resolution of the Query ( $Q\in {\mathbb{R}}^{H_{q}W_{q}\times C}$, where $H_{q}=W_{q}=7$ and C=256). For each stage s∈{1,2,3}, the Key and Value pairs ( $K_{s},V_{s}\in {\mathbb{R}}^{H_{s}W_{s}\times C}$) feature spatial resolutions ( $H_{s}\times W_{s}$) of 56×56, 28×28, and 14×14, respectively. The exact operation count for standard global cross-attention at stage *s* is calculated as shown in Equation (5):

(5) $${\text{FLOPs}}_{\text{global}}^{\left(s\right)}=4H_{q}W_{q}C^{2}+2H_{s}W_{s}C^{2}+2\left(H_{q}W_{q}\right)\left(H_{s}W_{s}\right)C$$

where the first two terms represent the linear projections and the final term accounts for the sequential matrix multiplications computing the attention map and aggregation. In contrast, our KGLA module restricts each query token’s interaction to a local sub-window of size *M_s_*× *M_s_*, where *M*_1_ = 8, *M*_2_ = 4, and *M*_3_ = 2. This local constraint effectively linearizes the quadratic token-to-token product, yielding a reduced stage operational count shown in Equation (6):

(6) $${\text{FLOPs}}_{\text{KGLA}}^{\left(s\right)}=4H_{q}W_{q}C^{2}+2H_{s}W_{s}C^{2}+2\left(H_{q}W_{q}\right)M_{s}^{2}C.$$

To quantify this structural reduction, the theoretical efficiency ratio *R_s_* for the core attention computation phase (excluding linear projections) is derived as shown in Equation (7):

(7) $$R_{s}=\frac{FLOPs_{attention\_global}^{\left(s\right)}}{FLOPs_{attention\_KGLA}^{\left(s\right)}}\approx \frac{2\left(H_{q}W_{q}\right)\left(H_{s}W_{s}\right)C}{2\left(H_{q}W_{q}\right)M_{s}^{2}C}=\frac{H_{s}W_{s}}{M_{s}^{2}}.$$

The accumulated attention computation across the three stages (excluding the shared linear projection operations) is reduced from 103.26M FLOPs for standard global cross-attention to only 2.11M FLOPs for the proposed KGLA, corresponding to a 49× reduction. Including the shared linear projection operations, the total computational cost is reduced from 0.681G FLOPs to 0.580G FLOPs. These analytical results demonstrate that KGLA effectively suppresses redundant cross-regional interactions while substantially improving computational efficiency.

### 3.5 Self-attention and emotion classification

After the local visual information has been aggregated via KGLA, the resulting multi-scale features are further processed to model global dependencies. A keypoint feature self-attention (11) layer is applied to the aggregated representations, enabling self-interaction among the features to fortify their global contextual relationships. For the final emotion classification, we design a query-based decoding head. We introduce an initial query vector initialized with zeros ( $Q_{init}\in {\mathbb{R}}^{1\times 256}$). Furthermore, this global query iteratively interacts with the output of the self-attention layer (which serves as the post-attention keypoints) through a series of three stacked attention blocks. It is worth noting that these stacked attention blocks operate utilizing the exact same MHCA formulation as the localized mechanism described above. By iteratively refining the query, the network extracts the most highly discriminative, high-order semantic representations for expression analysis. Finally, the optimized query is passed through a Multi-Layer Perceptron (MLP) to output the predicted emotion classification probabilities.

## 4 Experiments

In this section, we comprehensively evaluate the proposed approach. We first introduce the benchmark datasets utilized for facial expression recognition (FER). Subsequently, we detail the network implementation, general training protocols, and dataset-specific optimization strategies.

### 4.1 Datasets

**Figure 4:**
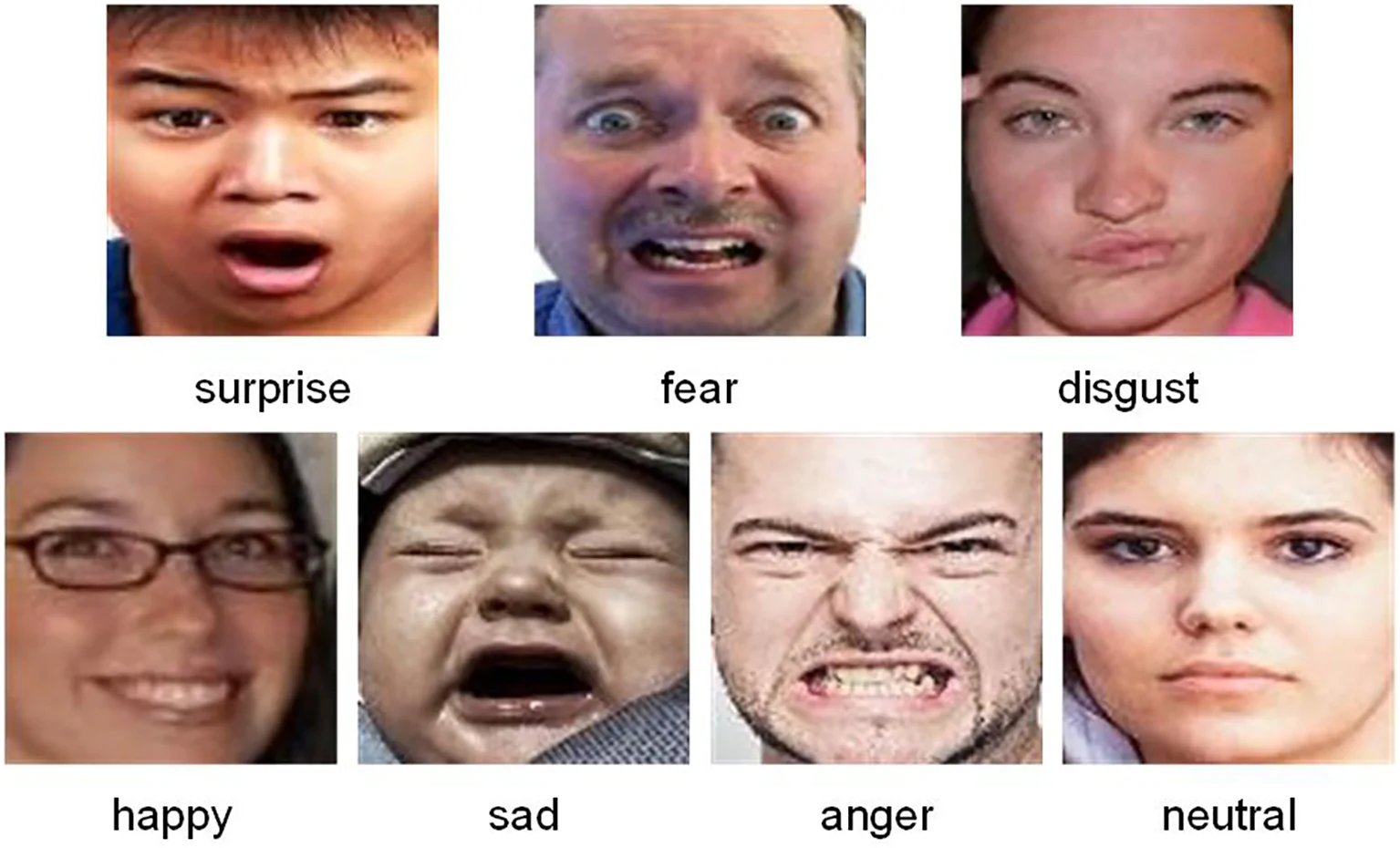
Representative samples from the RAF-DB dataset, illustrating the seven basic emotion categories: surprise, fear, disgust, happy, sad, anger, and neutral.

To rigorously assess the generalizability and robustness of our method, we conduct experiments on three widely adopted in-the-wild FER benchmarks:

- RAF-DB (1): The Real-world Affective Faces Database (RAF-DB) is a large-scale dataset containing diverse facial images. It provides annotations for seven basic emotion categories: neutral, happy, sad, surprise, fear, disgust, and anger. Representative examples of these seven expression categories are shown in **Figure 4**.
- AffectNet (2): As one of the most extensive publicly available datasets in the FER domain, AffectNet presents significant challenges due to its sheer scale and variance in the wild. We evaluate our model on both the widely used 7-class configuration and the 8-class configuration (which includes the additional “contempt” category).
- CAER-S (33): Derived from the Context-Aware Emotion Recognition (CAER) dataset, CAER-S focuses on static images and is categorized into seven distinct expression types corresponding to the primary human emotions.

### 4.2 Implementation details

Network Configuration. Following established paradigms in FER (9, 10), the image stream leverages an IR-50 (34) backbone pre-trained on the Ms-Celeb-1M dataset to extract visual representations. For the structural prior stream, we utilize MobileFaceNet (35) with frozen weights as our landmark detector. To facilitate our Keypoint-Guided Local Aggregation (KGLA) module, we extract latent feature maps from layers 3, and 6 of MobileFaceNet, which inherently provide hierarchical, multi-scale facial landmark features.

Training Configurations. The entire network is implemented using PyTorch and optimized end-to-end with the cross-entropy loss on a single NVIDIA RTX 4090 GPU. To accommodate the distinct data distributions, we specifically tune hyperparameters such as the base learning rate and data augmentation for each dataset, while maintaining general settings (e.g., optimizer, training epochs, and batch size) consistent. A comprehensive summary of the detailed training protocols across the four experimental settings is provided in **Table 1**.

**Table 1:** Implementation details and hyperparameter configurations across different datasets.

Configuration	RAF-DB	AffectNet (7 cls)	AffectNet (8 cls)	CAER-S
Optimizer	Adam	Adam	Adam	Adam
Training Epochs	200	200	200	200
Batch Size	128	128	128	128
Base Learning Rate	1e-5	1e-6	1e-6	1e-5
Weight Decay	1e-4	1e-4	1e-4	1e-4
LR Schedule (Exponential)	*γ* = 0.98	*γ* = 0.98	*γ* = 0.98	*γ* = 0.98
Drop Path	0→0.5	0→0.5	0→0.5	0→0.5
Data Augmentation	Flip, Erasing	Flip, Erasing	Flip, Erasing	Flip, Erasing
Random Erasing Scale	(0.02, 0.1)	*p* = 1, (0.05, 0.05)	*p* = 1, (0.05, 0.05)	*p* = 1, (0.05, 0.05)

### 4.3 Comparison with SOTA FER methods

To comprehensively evaluate the effectiveness of the proposed KGLA-Net, we compare it with a broad range of representative facial expression recognition (FER) methods on three widely used benchmarks: RAF-DB, AffectNet (7-class and 8-class), and CAER-S. Following common statistical evaluation practices, all results of KGLA-Net are reported as the Mean ± Standard Deviation (SD) over five independent runs with different random seeds.

Results on RAF-DB. As summarized in **Table 2**, KGLA-Net achieves an average top-1 accuracy of 92.43% ± 0.11%. This result achieves the highest accuracy among all compared methods. Compared with representative landmark-guided and Transformer-based approaches, including POSTER (9) (92.05%), HAM (54) (91.92%), and POSTER++ (10) (92.21%), the proposed method consistently achieves better recognition performance. Moreover, KGLA-Net also surpasses recent Mamba-based FER methods, including FERMam (31) (92.13%) and Mambaer (32) (91.69%). These results suggest that the proposed Keypoint-Guided Local Aggregation effectively exploits structural priors for cross-modal feature interaction, leading to more discriminative facial representations while maintaining stable performance across repeated runs.

**Table 2:** Comparison results with SOTA FER algorithms on RAF-DB and AffectNet datasets.

Methods	Year	RAF-DB (%)	AffectNet (7 cls) (%)	AffectNet (8 cls) (%)
PSR (36)	2020	88.98	63.77	60.68
DACL (37)	2020	87.78	65.20	–
VTFF (38)	2021	88.14	61.85	–
ARM (39)	2021	90.42	65.20	61.33
TransFER (7)	2021	90.91	66.23	–
EfficientFace (40)	2021	88.36	63.70	60.23
MA-Net (41)	2021	88.42	64.53	60.29
Zhang et al. (42)	2022	87.10	62.10	–
Meta-Face2Exp (43)	2022	88.54	64.23	–
EAC (44)	2022	90.35	65.32	–
AMP-Net (45)	2022	89.25	64.54	61.74
FER-CHC (46)	2023	90.06	66.31	–
FST-MWOS (47)	2023	90.38	63.33	–
SwinFace (48)	2023	90.97	–	–
MACN (49)	2023	90.16	63.12	–
Face2Nodes (50)	2023	91.02	66.69	–
MRAN (51)	2023	90.03	66.31	62.48
SqueezExpNet (52)	2023	80.65	–	–
SSA-ICL-ResNet18 (53)	2023	89.44	65.78	–
POSTER (9)	2023	92.05	67.31	63.34
HAM (54)	2024	91.92	66.97	63.82
Wang et al. (55)	2024	–	62.70	–
GSDNet (18)	2024	90.91	66.11	–
FS-DSN (19)	2025	88.82	–	–
POSTER++ (10)	2025	92.21	67.49	63.77
MM-Net (22)	2026	90.42	66.06	–
FERMam (31)	2026	92.13	66.38	61.45
Mambaer (32)	2026	91.69	66.77	–
Ours (KGLA-Net)	2026	92.43 ± 0.11	67.83 ± 0.09	64.01 ± 0.08

Results on AffectNet. The comparison results on the challenging AffectNet benchmark are summarized in **Table 2**. KGLA-Net achieves average accuracies of 67.83% ± 0.09% and 64.01% ± 0.08% on the 7-class and 8-class settings, respectively. Compared with recent state-of-the-art methods, including POSTER++ (10), FERMam (31), and Mambaer (32), KGLA-Net consistently achieves the best performance on both evaluation settings. These results further demonstrate the effectiveness of the proposed landmarkguided local aggregation strategy in learning robust facial representations under unconstrained real-world conditions.

Results on CAER-S. To further evaluate the generalization capability of the proposed framework, we conduct experiments on the context-aware CAER-S dataset. As shown in **Table 3**, KGLA-Net achieves an average accuracy of 93.20% ± 0.13%. Compared with recent representative methods, including HAM (54) (92.86%) and POSTER++ (10) (93.00%), KGLA-Net achieves the highest recognition accuracy. These results further demonstrate that the proposed landmark-guided local aggregation strategy generalizes well across different FER benchmarks while maintaining stable performance over repeated runs.

**Table 3:** Comparison results with SOTA FER methods on the CAER-S dataset.

Methods	Year	CAER-S (%)
DSN (56)	2018	75.19
CAER-Net-S (33)	2019	73.51
GRERN (57)	2020	81.31
EfficientFace (40)	2021	85.87
MA-Net (41)	2021	88.42
GLAMOR-Net (58)	2021	89.88
POSTER (9)	2023	92.73
SMResNet (59)	2023	88.52
HAM (54)	2024	92.86
POSTER++ (10)	2025	93.00
Ours (KGLA-Net)	2026	93.20 ± 0.13

To further characterize the statistical uncertainty of the reported results, we additionally report the 95% confidence interval (CI) of KGLA-Net on each benchmark. The confidence intervals are computed using the Student’s *t*-distribution over the five independent runs and are summarized in **Table 4**. As can be observed, KGLA-Net exhibits narrow confidence intervals across all datasets, indicating that the proposed framework achieves stable performance under different random initializations.

**Table 4:** Statistical uncertainty of KGLA-Net over five independent runs.

Dataset	Mean ± SD (%)	95% CI (%)
RAF-DB	92.43 ± 0.11	[92.29, 92.57]
AffectNet (7 cls)	67.83 ± 0.09	[67.72, 67.94]
AffectNet (8 cls)	64.01 ± 0.08	[63.91, 64.11]
CAER-S	93.20 ± 0.13	[93.04, 93.36]

The 95% confidence intervals are calculated using the Student’s *t*-distribution.

### 4.4 Comparison of computational efficiency

Balancing recognition accuracy with computational efficiency is crucial for deploying FER models in practical applications. Therefore, we compare the proposed KGLA-Net with representative FER methods in terms of the number of parameters (#Params), floating-point operations (#FLOPs), inference latency, and recognition accuracy on the RAF-DB and AffectNet (7-class) datasets. The results are summarized in **Table 5**. Transformer-based FER models generally suffer from the quadratic computational complexity of global self/cross-attention, i.e., *O*(*N*^2^*D*), where *N* denotes the spatial sequence length and *D* is the feature dimension. Consequently, representative methods such as TransFER (7) and POSTER (9) require relatively high computational costs (15.3G and 15.7G FLOPs, respectively). To improve computational efficiency, the proposed KGLA performs cross-modal interaction within landmark-guided local windows, reducing the attention complexity to *O*(*M*^2^*ND*), where *M* represents the local window size. Moreover, unlike POSTER++ (10), which performs cross-modal interaction using the complete landmark representation, KGLA aggregates visual features only within semantically corresponding local regions, thereby avoiding unnecessary cross-regional interactions. As shown in **Table 5**, KGLA-Net achieves a favorable balance between efficiency and recognition performance. The proposed model contains only 31.2M parameters and requires 7.2G FLOPs, both lower than POSTER++ (43.7M parameters and 8.4G FLOPs), while achieving higher recognition accuracy on both RAF-DB and AffectNet. Furthermore, KGLA-Net requires only 7.9 ms per image for inference, demonstrating that the proposed localized aggregation strategy improves not only theoretical computational complexity but also practical inference efficiency, making it suitable for resource-constrained FER applications.

**Table 5:** Comparison of #Params, #FLOPs, #Time, and accuracy with other representative methods.

Methods	#Params	#FLOPs	Time	RAF-DB (%)	AffectNet (7 cls) (%)
MA-Net (41)	63.5M	3.7G	–	88.42	64.53
DACL (37)	103.0M	1.9G	–	87.78	65.20
VTFF (38)	51.8M	–	–	88.14	61.85
FST-MWOS (47)	147.9M	33.82G	–	90.38	63.33
PACVT (60)	45.9M	–	–	87.18	59.76
TransFER (7)	65.2M	15.3G	–	90.91	66.23
DMUE (3)	78.4M	13.4G	–	89.42	63.11
SwinFace (48)	67.3M	23.8G	–	90.97	–
HAM (54)	69.63M	–	–	91.92	66.97
POSTER (9)	71.8M	15.7G	–	92.05	67.31
POSTER++ (10)	43.7M	8.4G	–	92.21	67.49
Ours (KGLA-Net)	31.2M	7.2G	7.9ms	92.48	67.87

### 4.5 Ablation study

To comprehensively evaluate the effectiveness of our proposed network designs, we conduct extensive ablation studies on the RAF-DB dataset. We systematically investigate the contributions of the core components, the impact of the multi-scale aggregation strategy, and the design choices for the final classification module.

#### 4.5.1 Effectiveness of key components

We first verify the individual contributions of our proposed modules: the FRM, the KGLA module, and our query-based classification head. To establish a baseline, we design a simple two-stream network without any cross-modal interaction. In this baseline, the final stage features of the image and keypoint streams are independently processed by Global Average Pooling (GAP), combined via element-wise addition, and passed to a linear layer for classification. As shown in **Table 6**, the baseline network only achieves an accuracy of 88.52%. The introduction of the FRM provides a performance boost to 89.65%, demonstrating that refining the initial features aids in constructing a better structural prior. When the core KGLA module is integrated to replace the simple feature addition, the accuracy significantly jumps to 91.83%. This substantial improvement proves that localized, keypoint-guided cross-attention effectively bridges the modality gap and captures fine-grained facial variations while suppressing background noise. Finally, replacing the standard pooling classifier with our query-based Classification Module yields the highest accuracy of 92.48%. This confirms that iteratively refining a global query extracts high-order discriminative semantics far better than naive pooling.

**Table 6:** Ablation study of the key components on the RAF-DB dataset. “Baseline” denotes the simple two-stream addition network.

Baseline	+ FRM	+ KGLA	+ query-based head	RAF-DB (%)
✓				88.52
✓	✓			89.65
✓	✓	✓		91.83
✓	✓	✓	✓	**92.48**

Bold values indicate the best-performing results.

#### 4.5.2 Visualization of feature refinement

To intuitively demonstrate the effectiveness of the proposed FRM and the quality of the generated structural priors, we visualize the spatial refinement score maps (Si) on representative samples from the dataset. First, we overlay the extracted heatmaps onto the original input images across different emotion categories (e.g., Happy, Sad, Surprise). The visualizations reveal that the FRM accurately assigns higher activation weights to expression-salient facial regions. For instance, in the “Happy” samples, the network heavily focuses on the raised cheeks (apple muscles) and the extended corners of the mouth. In the “Sad” and “Surprise” samples, the attention dynamically shifts to the furrowed brows, tense eye regions, and widened mouth. Conversely, identity-specific textures and irrelevant background areas receive significantly suppressed scores (indicated by cooler colors). This qualitative evidence explicitly confirms that the lightweight similarity scoring mechanism in the FRM distills pure emotional cues, providing a low-noise structural anchor to accurately guide the subsequent KGLA module.

#### 4.5.3 Impact of multi-scale aggregation in KGLA

A key innovation of our KGLA is the multi-scale aggregation of visual features guided by keypoint priors across different stages (Stage 1, Stage 2, and Stage 3). To validate this design, we ablate the KGLA combinations across different stages. As reported in **Table 7**, applying KGLA to only a single stage yields suboptimal results (e.g., 90.25% for Stage 1 and 91.10% for Stage 3). While lower stages (Stage 1) capture fine-grained textural details like wrinkles and micro-expressions, higher stages (Stage 3) abstract coarse structural semantics. Fusing multiple stages consistently improves performance. For instance, aggregating both Stage 2 and Stage 3 reaches 91.95%. The optimal performance (92.48%) is achieved only when all three stages are sequentially cascaded. This clearly demonstrates that a holistic multi-scale aggregation strategy is essential for capturing the full spectrum of expression-related muscular dynamics.

**Table 7:** Ablation study of KGLA aggregation across different stages on RAF-DB.

Stage 1	Stage 2	Stage 3	RAF-DB (%)
✓			90.25
	✓		90.68
		✓	91.10
✓	✓		91.44
	✓	✓	91.95
✓	✓	✓	92.48

#### 4.5.4 Design of the classification module

Finally, we analyze the design of the classification head. After the aggregated features pass through the keypoint feature self-attention layer, a decoding strategy is required. We compare our proposed querybased decoding head with two standard feature aggregation methods: Global Max Pooling (MaxPool) and Global Average Pooling (AvgPool) followed by a linear classification layer. **Table 8** illustrates the comparison. Standard MaxPool and AvgPool achieve 91.35% and 91.70%, respectively. In contrast, the proposed query-based decoding head iteratively interacts with the post-attention keypoint features through an initialized global query, preserving more informative structural representations and improving the recognition accuracy to 92.48%.

**Table 8:** Comparison of different classification head designs on RAF-DB.

Classification head design	RAF-DB (%)
MaxPool	91.35
AvgPool	91.70
Query-based	92.48

#### 4.5.5 Ablation on query initialization strategy

To evaluate the empirical stability of the proposed query-based classification head, we conduct an ablation study on the initialization strategy of the initial query vector *Q*_init_ ∈ R^1×256^. We compare our zero-initialization strategy against Xavier random initialization configured across five distinct random seeds on the RAF-DB dataset. As shown in **Table 9**, the experimental results show that the zero-initialization strategy yields a top-1 accuracy of 92.48%, while the five randomly initialized models deliver accuracies tightly clustered between 91.92% and 92.51%. Among the five baseline configurations, four produce slight variations below our design and one lands marginally above it. The minimal performance variance across all seeds indicates that initializing the initial query to zero does not induce optimization instability or compromise the convergence of the network, validating it as a reliable and deterministic alternative to stochastic initialization.

**Table 9:** Ablation study on query initialization strategies on the RAF-DB.

Initialization strategy	Random seed	Top-1 acuracy (%)
Xavier Random Initialization	10	91.92
Xavier Random Initialization	42	92.15
Xavier Random Initialization	100	92.30
Xavier Random Initialization	512	92.37
Xavier Random Initialization	2026	92.51
Zero Initialization (Ours)	N/A	92.48

### 4.6 Comparison with different efficient cross-attention strategies

To further verify the effectiveness of the proposed Keypoint-Guided Local Aggregation (KGLA), we compare it with two representative cross-attention strategies while keeping all other network components unchanged. Specifically, we consider (1) Global Cross Attention, where the Local Keypoint Feature directly attends to the full-resolution image features, and (2) Spatial Reduction Cross Attention (SRA), which follows the spatial reduction strategy of PVT (27) by applying average pooling to the image features before cross-attention computation. As shown in **Table 10**, replacing KGLA with either Global Cross Attention or Spatial Reduction Cross Attention leads to inferior recognition performance. Although SRA reduces the computational burden by compressing image features before attention computation, the spatial reduction inevitably weakens fine-grained structural guidance. In contrast, KGLA performs cross-modal aggregation within corresponding local regions, allowing landmark priors to guide visual feature learning more effectively. These results demonstrate that the performance gain originates from the proposed localized keypoint-guided aggregation mechanism rather than simply adopting a lightweight attention strategy.

**Table 10:** Comparison of different cross-attention strategies on RAF-DB.

Cross-attention strategy	Accuracy (%)
Global Cross Attention	92.06
Spatial Reduction Cross Attention (PVT)	91.44
KGLA (Ours)	92.48

#### 4.6.1 Visualization of keypoint-guided local aggregation

To provide an intuitive understanding of the proposed KGLA mechanism, we visualize the attention distributions generated by the image stream under different query landmarks. Two representative facial keypoints (the right eye and mouth corner) are selected as Query locations. For each Query, we compare the attention distributions produced by the conventional global cross-attention and the proposed Keypoint-Guided Local Aggregation. The global attention maps exhibit relatively dispersed responses over the entire face, assigning non-negligible attention weights to multiple irrelevant facial regions. Such globally distributed interactions inevitably introduce redundant contextual information and increase computational complexity. In contrast, the proposed KGLA explicitly restricts feature aggregation to a small landmark-guided local neighborhood. The responses become highly concentrated around semantically corresponding facial muscles and texture patterns, while suppressing unrelated regions. For example, the eye Query primarily focuses on the eyebrow–eye area, whereas the mouth Query mainly aggregates features around the mouth contour. This localized aggregation enables more discriminative feature extraction with substantially reduced computational cost, demonstrating that the learned attention regions are well aligned with meaningful facial muscle movements. The qualitative visualization is also consistent with the quantitative results reported in **Table 10**. Despite performing global feature interaction, Global Cross Attention achieves lower recognition accuracy than the proposed KGLA, suggesting that localized landmark-guided aggregation is more effective than globally distributed attention for cross-modal feature learning.

### 4.7 Robustness to degraded landmark quality

Since KGLA-Net performs cross-modal aggregation by using the structural representations extracted from the Landmark Backbone as queries, the quality of the structural prior directly affects the effectiveness of Keypoint-Guided Local Aggregation. To evaluate the robustness of the proposed framework under degraded landmark quality, we conduct an additional robustness experiment by randomly masking different proportions of the input facial image before it is processed by the Landmark Backbone. Specifically, randomly selected square patches are replaced with zero values, resulting in masking ratios of 10%, 20%, 30%, 40%, and 50%, while the Image Backbone remains unchanged. This strategy progressively degrades the structural representations extracted from the Landmark Backbone and simulates practical situations such as partial facial occlusion, inaccurate landmark localization, and missing facial cues. The quantitative results are summarized in **Table 11**. All results are reported as the mean and standard deviation over five independent runs. As shown in

**Table 11:** Robustness evaluation under different masking ratios on RAF-DB. Results are reported as the mean ± standard deviation over five independent runs.

Mask ratio	0%	10%	20%	30%	40%	50%
Accuracy (%)	92.43 ± 0.11	92.16 ± 0.15	91.58 ± 0.20	90.47 ± 0.28	88.21 ± 0.37	85.36 ± 0.51

**Table 11**, the recognition accuracy decreases progressively as the masking ratio increases, confirming that reliable structural priors are essential for effective cross-modal feature aggregation. Under mild degradation (10%–20%), the performance drops only slightly, indicating that the proposed Feature Refinement Module is able to extract stable structural cues even when a limited portion of facial information is missing. As the masking ratio further increases to 30%–50%, the degradation becomes more pronounced because the Landmark Backbone can no longer provide sufficiently accurate structural guidance for Keypoint-Guided Local Aggregation. Nevertheless, the performance decreases in a gradual rather than abrupt manner, demonstrating that the proposed multi-scale feature refinement and localized aggregation strategy remain effective under imperfect landmark estimation. These results indicate that KGLA-Net maintains reasonable robustness to degraded landmark quality while also verifying the importance of accurate structural guidance for the proposed cross-modal aggregation mechanism.

### 4.8 Cross-dataset generalization

To further evaluate the generalization capability of KGLA-Net under unseen data distributions, we additionally conduct cross-dataset experiments between RAF-DB and AffectNet (7-class). Specifically, the model is trained on one dataset using the standard training protocol and directly evaluated on the other dataset without any fine-tuning or domain adaptation. The quantitative results are summarized in **Table 12**. As expected, the recognition accuracy decreases considerably compared with the conventional within-dataset evaluation due to the substantial domain gap between RAF-DB and AffectNet. Nevertheless, the proposed KGLA-Net achieves 56.82% when trained on RAF-DB and tested on AffectNet, and 74.35% in the reverse setting. The latter achieves higher performance because AffectNet contains substantially larger training data and richer appearance variations, enabling the model to learn more transferable facial representations. These preliminary results demonstrate that KGLA-Net maintains a reasonable degree of cross-dataset generalization, while further improving domain robustness through domain adaptation or domain generalization techniques remains an important direction for future research.

**Table 12:** Cross-dataset evaluation between RAF-DB and AffectNet (7-class).

Training dataset	Testing dataset	Accuracy (%)
RAF-DB	AffectNet (7 cls)	56.82 ± 0.34
AffectNet (7 cls)	RAF-DB	74.35 ± 0.27

## 5 Conclusion and future work

To overcome the quadratic computational overhead and spatial redundancy typical of global cross-attention mechanisms, we proposed an efficient FER framework (KGLA-Net). By strictly aligning structural keypoint priors with visual features within dynamically shrinking local windows, our network distills expressionrelevant muscular dynamics from complex backgrounds with linear computational complexity. Furthermore, the cascaded query mechanism and the stacked query-based decoding head ensure progressive feature enhancement and the optimal extraction of high-order semantic representations. Extensive experiments across the RAF-DB, AffectNet, and CAER-S benchmarks demonstrate that KGLA-Net achieves advanced accuracy.

Despite its superior performance, KGLA-Net inherently relies on the quality of the extracted facial landmarks. In severe real-world occlusion scenarios (e.g., heavy objects, thick glasses, or masks), the structural prior may be degraded, which could subsequently compromise the local aggregation process. In future work, we plan to address this limitation by integrating KGLA-Net with Masked Image Modeling (MIM) paradigms to learn robust, occlusion-invariant representations. Additionally, exploring a unified, end-to-end framework that jointly optimizes landmark detection and facial expression recognition in a mutually beneficial manner will be a promising direction to further push the boundaries of FER in the wild.

## Data Availability

The original contributions presented in the study are included in the article/supplementary material. Further inquiries can be directed to the corresponding author.
